# Prevalence and Impact of Anxiety and Depression on Type 2 Diabetes in Tunisian Patients over Sixty Years Old

**DOI:** 10.1155/2013/341782

**Published:** 2013-06-19

**Authors:** Jawaher Masmoudi, Rahma Damak, Hela Zouari, Uta Ouali, Anouar Mechri, Nouri Zouari, Abdelaziz Jaoua

**Affiliations:** ^1^Department of Psychiatry A, University Medical Center Hédi Chaker, 3000 Sfax, Tunisia; ^2^Department of Functional Exploration and Metabolic Diseases, University Medical Center Habib Bourguiba, 3000 Sfax, Tunisia; ^3^Department of Psychiatry, University Medical Center Fattouma Bourguiba, 5000 Monastir, Tunisia

## Abstract

*Objectives*. To estimate the prevalence of anxiety and depression using the Hospital Anxiety and Depression Scale (HADS) in a population aged over sixty years with type 2 diabetes and to study the impact of anxiety and depression on glycemic balance and disease outcome. *Results*. The prevalence of anxiety and depression in the 62 subjects included in the study was, respectively, 40.3% and 22.6%. We found a relationship between these disorders and complicated diabetes. The subjects having an imperfectly balanced diabetes had a higher average anxiety score than those having a good glycemic control (9.1 ± 4.2 versus 6.5 ± 3.1; *P* = 0.017). No relationship was found between diabetes balance and depression. *Conclusion*. Association between anxiety and depressive disorders and diabetes is frequent and worsens patients' outcome, in terms of diabetes imbalance as well as in terms of diabetic complications. Our study shows that there is need for physicians to detect, confirm, and treat anxiety and depressive disorders in elderly diabetic patients.

## 1. Introduction 

In Tunisia, the proportion of individuals over 60 years is increasing, and it reached 9% in 2004 [1]. 

Diabetes is a major public health problem in Tunisia. Its prevalence has increased steadily and is currently about 9.9% [2]. Prevalence increases with age (11.9% for 60 years or older) [3, 4]. In addition, depression and anxiety, at the top of mental disorders list, mainly remain undiagnosed [5, 6], in particular in the elderly [7, 8] and consequently untreated [9]. Studying the link between diabetes and depressive as well as anxiety disorders in elderly subjects is useful for several reasons. First, diabetes as well as depression constitutes a major source of functional incapacity, and thus of loss of autonomy, in the elderly population [10]. Second, the association of these two pathologies in the same person worsens total health outcome [11] and quality of life [6, 12]. Finally, this association increases health care expenditures [11] and mortality [13]. The aim of this study is to estimate the prevalence of anxiety and depression in a population of elderly subjects aged over sixty suffering from type 2 diabetes and to study the impact of anxiety and depression on glycemic control and the evolution of the disease.

## 2. Methods 

We undertook a descriptive and analytical cross-sectional study on 62 patients who have consulted during September and October 2010 at the Department of Functional Explorations and Metabolic Diseases of Habib Bourguba University Hospital, Sfax, Tunisia. Recruitment was carried out on an ad hoc basis. 

The patients included were those who had agreed to take part in the investigation and who were 60 years old or over. We used this age limit because under Tunisian law, 60 is the retirement age.

Exclusion criteria were the incapacity to answer the questions because of cognitive deterioration and refusal to take part in the study. 

We used a semistructured questionnaire composed of sociodemographic and professional characteristics which was administered by the same investigator. We collected the clinical data relating to diabetes (duration, personal medical history: dyslipidemia, cardiac and vascular disorders, obesity, and diabetic complications such as macroangiopathy, nephropathy, peripheral neuropathy, and erectile dysfunction) and therapeutic data (physical exercise, diet, regular followup, treatment compliance). The balance of diabetes was assessed by the last rate of glycated hemoglobin (HbA1) and/or fasting glycaemia. Diabetes was considered as balanced if HbA1 <7% and/or fasting glycaemia ≤6 mmol/L. 

To evaluate the mood and anxiety state of subjects, we used the Hospital Anxiety and Depression Scale (HADS), a self-assessment scale that has been developed and found to be a reliable instrument for detecting states of depression and anxiety in the setting of a hospital medical outpatient clinic. The anxiety and depressive subscales are also valid measures of severity of the emotional disorder. It is suggested that the introduction of HADS into general hospital practice would facilitate the large task of detection and management of emotional disorder in patients under investigation and treatment in medical and surgical departments. The thresholds for pathological anxiety and depression were 10 on each of the two subscales (A for anxiety and D for depression) of HADS [14]. We used a validated Arabic version of this questionnaire [15]. The subjects having an elevated score (≥10) were referred to the psychiatric outpatient clinic for specialized evaluation and treatment.

Data analysis was made using the software SPSS for Windows version 11. For the quantitative variables, we calculated averages and standard deviations. We then compared the sub groups: anxious: *A* ≥ 10 to nonanxious: *A* < 10 and depressed: *D* ≥ 10 to not depressed by chi-square test of Pearson or the Fisher exact test as well as Student's “*t*” test of. Statistical significance was designated as *P* less than 0.05.

## 3. Results 

### 3.1. Sociodemographic Characteristics

Sociodemographic characteristics are summarized in Table 1. In our sample, a female predominance was observed. The average age of included subjects was 66.8 years (standard deviation = 4.8) with patients aged 60 years minimum/80 years maximum. More than two-thirds of the subjects were married. The socioeconomic level was qualified as average in more than 40% of the participants. A little less than three quarters of the subjects had no professional qualifications.

### 3.2. Clinical and Therapeutic Characteristics

The various clinical and therapeutic characteristics are presented in Table 2. The average age of diabetes onset was 54.5 years (standard deviation = 9.4), ranging from 25 to 74 years. The average duration of the diabetes was 12.4 years (standard deviation = 8.1), ranging from 0 to 36 years. The near total of included subjects (90.3%) had a somatic comorbidity associated with the diabetes. Hypertension was the most frequent comorbidity. More than two-thirds of the sample had poor glycemic control. The average fasting glycaemia was 9.4 mmol/L (standard deviation = 4.4 mmol/L) and average HbA1 was 9% (standard deviation = 2.1%). Fifty-seven subjects affirmed having a good compliance with treatment. 

### 3.3. Prevalence of Anxiety and Depression

The prevalence of anxiety and depression was, respectively, 40.3% and 22.6%. The average score of anxiety was 8.3 ± 4.4. The average score of depression was 6.1 ± 4.3 (Table 3). 

### 3.4. Relationship between Anxiety and Depression and Sociodemographic Characteristics

According to the HADS scale, women were significantly more anxious than men. The subjects with no professional qualifications scored higher on the anxiety subscale than did subjects with a profession. In our study, we did not find a link between depression and the different sociodemographic variables (Table 4).

### 3.5. Relationship between Anxiety and Depression and Diabetes Clinical Characteristics

In our study, we did not note a statistically significant correlation between duration of diabetes and anxiety and depression (13.1 ± 7.1 years in anxious subjects versus 11.9 ± 8.7 years in nonanxious subjects; *P* = 0.59 and 13.7 ± 8.3 years in depressed subjects versus 12 ± 8 years in nondepressed subjects, *P* = 0.48). When we studied the link between anxiety/depression and diabetes somatic comorbidities, we found that the average anxiety score of the subjects having a somatic comorbidity was higher, except for subjects having obesity as a comorbidity. However, this correlation was statistically significant only for hypertension. A similar correlation was not found when we studied the relationship between depression and somatic comorbidity. As to glycaemic control, average anxiety score was significantly higher amongst patients whose diabetes was badly managed. Nevertheless, there was no relationship between depression and diabetes balance in our sample. We noted that patients with complicated diabetes had a significantly higher average anxiety score (9.4 ± 3.9 versus 6.3 ± 3.7; *P* = 0.004). Indeed, anxious subjects had more diabetes complications, especially nephropathy and retinopathy. Subjects suffering from peripheral neuropathy, and retinopathy had a higher depression average score (Table 5).

Logistic regression analysis showed that anxiety was principally related to female gender (*P* = 0.008) and to the presence of nephropathy (*P* = 0.032). On the other hand, depression was correlated with the two variables: peripheral neuropathy (*P* = 0.004) and retinopathy (*P* = 0.05)

### 3.6. Relationship between Anxiety, Depression, and Diabetes Treatment Characteristics

In our study, we did not find a correlation between diabetes treatment characteristics, anxiety, and depression.

## 4. Discussion

The prevalence of anxiety and depression, according to HADS scores, was, respectively, 40.3% and 22.6% in our sample of elderly diabetic subjects.

In the literature, the prevalence of depression in elderly type 2 diabetics varies from 4.5% [11] to 17% [10]. Contrary to depression, there are few studies [16–18] examining anxiety in type 2 diabetics, particularly in older subjects. A study on a sample of 1,066 elderly type 2 diabetics aged 69 to 74 years [17], showed an average anxiety score of 5.7 ± 3.9 on the HADS scale and an average depression score of 3.9 ± 2.9. The anxiety and depression scores of the patients in our sample were higher. This difference could be explained by our sample being derived from subjects consulting at a hospital. Indeed, diabetic subjects who consulted at the hospital were generally referred there due to difficulties during treatment in first line structures. Consequently, they might have had a higher risk to present anxiety and depression. That is why it seems to be important to detect and treat these disorders as early as possible. 

The relationship between mood disorders and diabetes is bidirectional [10, 13, 19]. Several explanations are proposed that are inevitably interconnected; mood disorders are associated with an unhealthy lifestyle (tobacco abuse, little or no exercise, and excessive caloric intake) [20]. Depression is also related to obesity, which in turn is responsible for intolerance to glucose. Furthermore, depression is associated with physiological abnormalities, including the activation of the hypothalamus-pituitary-adrenal axis, the sympathetic nervous system, and the proinflammatory cytokines [18, 20], which can induce a resistance to insulin, and thus increase the risk of diabetes. Diabetes might increase the risk of depression and anxiety because of feelings of threat and loss related to the announcement of the diagnosis and the need to make lifestyle changes [11]. Finally, the association between mood disorders and diabetes can be partly explained by the existence of comorbidities [3, 18]. 

In our study, patients having a poorly controlled diabetes presented a significantly higher average anxiety score (*P* = 0.017). Indeed, these patients might be worried about complications related to their poor glycemic control. In addition, anxiety could deteriorate glycemic balance via adrenergic hyperactivity [21, 22]. We did not find a relationship between depression and diabetes balance; this has also been the case in several other studies on the subject [13, 23, 24]. 

In our study, subjects having high scores of anxiety and depression had significantly more complications of nephropathy, peripheral neuropathy and retinopathy. Indeed, depressed and anxious subjects are less likely to conform to recommendations concerning their diabetes that could affect glycemic control and consequently might impact the outcome of the disease [11, 13, 16]. Moreover, subjects suffering from diabetes are exposed to continuous stress generated by these complications, which may worsen or precipitate depression [11, 13, 18, 19]. Lastly, depression might constitute an etiopathologic factor responsible for the development of micro and macroangiopathy via pathophysiological modifications induced by deterioration of cellular immunity (diminished proliferative response of lymphocytes and reduction of NK activity) [11]. 

## 5. Limits of the Study

Although diabetes has a high prevalence in elderly subjects, our sample size was small. The reasons for this are varied. Our exclusion criteria left out a large proportion of patients, specifically those who were illiterate, those with cognitive impairments, and those who refused to join the study. Furthermore, the majority of diabetic patients in Tunisia are treated in first line health care facilities (private and public general practitioners). Thus, the hospital population represents only a part of the patients, most often those who had a diabetes which was difficult to manage. 

Our study has a cross-sectional design; therefore, a causal relationship between clinical variables related to diabetes and symptoms of anxiety and depression cannot be established. 

We used a psychometric scale, rather than a structured interview, to evaluate anxiety and depression. However, this instrument is frequently used as a screening tool in units for somatic diseases in order to detect psychiatric disorders and refer patients to a more specialized consultation. The interest of the HADS scale lies in the fact that it does not take into account the physical dimension of psychiatric symptoms, which could be a source of confusion. Indeed, symptoms which could be attributed to physical diseases such as insomnia, tiredness, headaches, and disturbances of appetite were omitted to avoid false positive findings amongst people with somatic diseases. 

Another limit to the study is the nature of the studied population. As was mentioned above, patients consulting at a hospital might have a higher prevalence of anxiety and depression than the patients consulting in first line facilities. 

## 6. Conclusion 

Diabetes, anxiety, and depression, are frequent pathologies, and each constitutes a public health problem in Tunisia. These pathologies are known to be more frequent in elderly people. Whereas diabetes is easy to detect and diagnose, this is not always the case with anxiety and mood disorders. According to recent estimates, more than three quarters of these psychiatric pathologies might not be detected. Several factors might explain this: the frequent expression of depression through somatic symptoms in the elderly, the tendency to attribute psychical suffering in the elderly to physical symptoms only the fear of being stigmatized as a psychiatric patient. Our results, in major part, are comparable to those of the literature and confirm the high prevalence of anxiety and depression in elderly subjects suffering from diabetes. The causal link between these two conditions seems to be bidirectional. In any case and according to our results, the association of these two types of pathologies impairs global outcome in terms of a greater diabetes imbalance and more frequent complications. Thus the need to detect, confirm, and treat anxiety and depressive disorders in elderly diabetic subjects. This will only be possible through close collaboration between geriatrists, diabetologists, nutritionists, and psychiatrists. 

## Figures and Tables

**Table 1:** Sociodemographic characteristics of the sample.

Sociodemographic characteristics	Number (%)
Sex	
Male	16 (25.8)
Female	46 (74.2)
Marital status	
Single	0 (0)
Married	40 (64.5)
Divorced	1 (1.6)
Widowed	21 (33.9)
Socioeconomic level	
Low	26 (42)
Medium	25 (40.3)
High	11 (17.7)
Resides	
With family	42 (88.7)
Alone	20 (11.3)
Profession	
No professional qualifications	45 (72.6)
Retired	15 (24.2)
Active	2 (3.2)

**Table 2:** Clinical and therapeutic characteristics of the sample.

Clinical and therapeutic characteristics	Number (%)
Somatic comorbidity	
Hypertension	45 (72.6)
Dyslipidemia	31 (50)
Heart disease	15 (24.2)
Obesity	13 (21)
Dysthyroidism	8 (12.9)
Balanced diabetes	
Yes	42 (67.7)
No	20 (32.3)
Diabetes complications	
Macro angiopathy	25 (40.3)
Peripheral neuropathy	24 (38.7)
Retinopathy	22 (35.5)
Nephropathy	8 (12.9)
Erectile dysfunction	3 (18.7)
Treatment compliance	
Yes	57 (91.1)
No	5 (8.9)
Follow a balanced diet	
Yes	37 (59.7)
No	25 (41.3)
Practice of physical activity	
Yes	19 (30.6)
No	45 (69.4)
Consultation mode	
Regular	60 (96.8)
Irregular	2 (3.2)

**Table 3:** Distribution of prevalence and average scores of anxiety and depression.

	Average score (%)
*A* ≥ 10	12.4 ± 2.4 (40.3)
*A* < 10	5.5 ± 2.1 (59.7)

*D* ≥ 10	12.2 ± 3.6 (22.6)
*D* < 10	4.4 ± 2.7 (77.4)

*A* ≥ 10: anxious; *A* < 10: not anxious; *D* ≥ 10: depressed; *D* < 10: not depressed.

**Table 4:** Relationship between sociodemographic characteristics, anxiety, and depression.

	*A* ≥ 10/*A* < 10	*P*	*D* ≥ 10/*D* < 10	*P*
Sexe M/F	2/23; 14/23	**0.008**	5/9; 11/37	0.36
Marital status				
Married	14/26	0.29	7/33	0.10
Divorced	0/1		1/0	
Widowed	11/10		6/15	
Profession				
Active	1/1	**0.009**	0/2	0.15
Retired	1/14		1/14	
No professional qualifications	23/22		13/32	
Resides				
Alone	5/2	0.07	1/6	0.57
With family	20/37		13/42	
Socioeconomic level				
Low	13/14	0.40	6/20	0.45
Medium	8/17		7/18	
High	4/7		1/10	

*N*: number of patients; M/F: male/female; *A* ≥ 10: anxious; *A* < 10: not anxious; *D* ≥ 10: depressed; *D* < 10: not depressed.

**Table 5:** Clinical and therapeutic characteristics of diabetes according to depression and anxiety average scores.

Clinical and therapeutic characteristics	AAS (SD)	*P*	DAS (SD)	*P*
Balanced diabetes				
Yes	6.5 (3.1)	**0.017**	5 (3.1)	0.14
No	9.1 (4.2)		6.7 (4.7)	
Comorbidity type				
Dyslipidemia				
Yes	9.1 (4)	0.14	6.5 (4.6)	0.47
No	7.5 (4)		5.7 (4.1)	
Heart disease				
Yes	9 (4.6)	0.47	7.6 (4.8)	0.15
No	8.1 (3.9)		5.7 (4.1)	
HTA				
Yes	9 (4.1)	**0.03**	6.5 (4.6)	0.24
No	6.5 (3.6)		5.1 (3.4)	
Obesity				
Yes	7.5 (4)	0.43	6.9 (5.3)	0.49
No	8.5 (4)		5.9 (4.1)	
Dysthyroidism				
Yes	8.2 (3.7)	0.94	5.1 (2.8)	0.47
No	8.3 (4)		6.3 (4.5)	
Complication type				
Macroangiopathy				
Yes	8.9 (3.8)	0.33	6.9 (4.5)	0.27
No	7.9 (4.3)		5.6 (4.2)	
Nephropathy				
Yes	**11.3** (3.2)	**0.024**	8.1 (4.7)	0.18
No	7.8 (4)		5.8 (4.3)	
Retinopathy				
Yes	**10.3** (4.1)	**0.004**	7.9 (4.6)	**0.017**
No	7.2 (3.7)		5.2 (3.9)	
Peripheral neuropathy				
Yes	9.4 (3.9)	0.10	8.1 (5.4)	**0.005**
No	7.6 (4.1)		4.9 (3)	
Erectile dysfunction				
Yes	7.3 (3.5)	0.67	7.3 (2.3)	0.64
No	8.3 (4.1)		6.1 (4.4)	

AAS: anxiety average score; DAS: depression average score; (SD): standard deviation.
